# Therapeutic drug monitoring of phenytoin and valproic acid in critically ill patients at Windhoek Central Hospital, Namibia

**DOI:** 10.4102/ajlm.v11i1.1628

**Published:** 2022-07-21

**Authors:** Bonifasius S. Singu, Helen Morrison, Lydia Irengeya, Roger K. Verbeeck

**Affiliations:** 1 School of Pharmacy, Faculty of Health Sciences, University of Namibia, Windhoek, Namibia

**Keywords:** phenytoin, valproic acid, critically ill patients, therapeutic drug monitoring, unbound concentration

## Abstract

**Background:**

Phenytoin and valproic acid, anticonvulsants, have a low therapeutic index and are highly plasma protein bound, mainly to albumin. Hypoalbuminaemia is common in critically ill patients and increases the unbound drug concentration. Thus, monitoring unbound rather than total plasma drug concentrations is recommended to optimise the dosing of these drugs.

**Objective:**

This retrospective study determined unbound plasma concentrations of phenytoin and valproic as a more accurate value of drug levels than total plasma drug concentrations.

**Methods:**

Total plasma concentrations were retrieved for 56 Intensive Care Unit patients for phenytoin and 93 for valproic acid. Total drug concentrations were converted to unbound concentrations using a serum albumin-based normalising equation.

**Results:**

Total phenytoin plasma concentration was below (41.1% of patients), within (46.4%) or above (12.5%) the therapeutic range (10 μg/mL – 20 μg/mL). However, the predicted unbound plasma concentration of phenytoin was above the therapeutic range (1 μg/mL – 2 μg/mL) in the majority of patients (57.1%). For valproic acid, the total plasma concentration of most patients (87.1%) was below the therapeutic range (50 μg/mL – 100 μg/mL); among remaining patients (12.9%), it was within the therapeutic range. In the majority of patients (91.4%), the predicted unbound plasma concentration of valproic acid was between 2.5 μg/mL and 20 μg/mL.

**Conclusion:**

The usefulness of monitoring the total phenytoin or valproic acid levels for dose optimisation is limited as it is an inaccurate indicator of a patient’s drug therapeutic state. Thus, the unbound plasma drug concentrations should be quantified experimentally or predicted in resource-limited settings.

## Introduction

The incidence of seizures in North American general intensive care units (ICUs) was reported in 2013 to range from 3.3% to 34.0%.^1^ Risk factors of seizures include head trauma, stroke, brain tumour, infections, hypoglycaemia, electrolyte abnormalities, and drug overdose. Phenytoin and valproic acid are anticonvulsants frequently used to prophylax or treat seizures in critically ill patients.^2,3^ However, both drugs have a low therapeutic index; thus, drug monitoring is required to minimise the risk of toxicity and optimise therapeutic efficacy.^4,5,6^ Following oral administration, both drugs are well-absorbed, highly bound to plasma albumin and eliminated by metabolism.^2,4,5^ The cytochrome P450 2C9 and 2C19 enzymes metabolise phenytoin to the inactive hydroxy-phenytoin, following first-order kinetics. However, at therapeutic concentrations, the rate of phenytoin metabolism approaches saturation and hence shows capacity-limited elimination (zero-order kinetics).^5^ Metabolism of valproic acid proceeds mainly by glucuronidation (50.0%) and β- and ω-oxidation (40.0%) and, to a much lesser extent, by cytochrome P450-catalysed oxidation (10.0%).^6^ The β-oxidation of valproic acid has been reported to be saturable and subject to autoinduction.^6^

The pharmacokinetic behaviour of phenytoin and valproic acid is complicated because their elimination kinetics are nonlinear. In addition, the pharmacokinetics of both drugs show a high degree of interindividual variability as a result of pharmacogenetic differences in enzyme and transporter activities, as well as high interpatient variability in plasma protein binding, in the case of valproic acid.^7,8,9^ As a result of this high interpatient variability, there is a poor correlation between the dose of these drugs and patient plasma concentrations. Hence, monitoring patients’ phenytoin or valproic acid plasma concentrations is common, even in critically ill patients.^2,7^

Phenytoin and valproic acid are highly bound to serum albumin (≥ 90%). The binding of these drugs depends on the drug plasma and albumin concentrations and the presence of other albumin-binding substances that may compete for binding sites with these drugs.^10^ Thus, hypoalbuminaemia, a condition in which concentrations of the plasma protein albumin are lower than 35 g/L, increases the unbound proportion of these drugs because of the decreased albumin (binding sites) concentrations.^11,12,13^ Hence, monitoring total plasma concentrations of phenytoin and valproic acid hypoalbuminaemia patients, such as critically ill patients, as the clinical indicator for therapeutic efficacy and safety, is not as reliable as utilising the unbound concentrations.^14,15^ Since it is only the unbound drug that can cross biological membranes and cause pharmacological action through interaction with the drug target, the therapeutic range and dosage individualisation in hypoalbuminaemia patients should be informed by the unbound plasma drug concentrations to prevent toxicity or therapeutic failure.^10,12,16,17^ Unbound plasma concentrations can be determined by equilibrium dialysis and ultrafiltration. However, the use of unbound plasma concentrations in clinical practice is limited because it is time consuming and costly. Therefore, equations have been formulated to estimate the unbound concentrations of phenytoin and valproic acid using a patient’s measured total drug plasma concentration and serum albumin level.^5,18,19,20,21,22^ The therapeutic range for the total phenytoin plasma concentration is 10 μg/mL to 20 μg/mL, and 1 μg/mL to 2 μg/mL for the unbound phenytoin plasma concentration.^4,5^ For valproic acid, the therapeutic range for the total plasma drug concentration is 50 μg/mL to 100 μg/mL; for the unbound plasma concentration, a therapeutic range of 2.5 μg/mL to 20 μg/mL has been suggested.^4,5^

The Windhoek Central Hospital in Windhoek (Namibia) conducts therapeutic phenytoin and valproic acid monitoring in critically ill patients in the ICU facility by measuring only total plasma concentrations of the drugs. This study aimed to retrospectively estimate unbound plasma concentrations as a more accurate measure of the therapeutic plasma levels of two drugs.

## Methods

### Ethical considerations

The Research and Ethics committees of the Ministry of Health and Social Services, the Republic of Namibia, approved this study; reference numbers HM2019 and LNI2019. Data were retrieved from patient record archives; no patients were required to participate in the data collection procedure of this study, and the ethics committee waived the requirement to obtain consent from patients before accessing their records. Furthermore, names were excluded from the data collected for this study to protect patients’ privacy.

### Patient population and data collection

This retrospective analysis utilised data from the ICU of Windhoek Central Hospital, Namibia. Records of patients admitted between January 2013 and July 2019 were reviewed. Patients who received phenytoin or valproic acid while at the ICU and whose anticonvulsant plasma concentrations and serum albumin levels had been recorded were included in the study. The following information was extracted from the records: age, gender, diagnosis, total plasma concentrations of phenytoin or valproic acid, serum albumin concentration, and concomitant medication. Serum albumin concentrations were measured at the Namibia Institute of Pathology using the bromocresol purple method (cobas^®^ 6000 Analyser, Roche Diagnostics International, Rotkreuz, Switzerland). The total plasma concentrations of phenytoin and valproic acid were measured by fluorescence polarisation immunoassay (Abbott Architect i2000, Abbott Laboratories, Chicago, Illinois, United States).

### Data analysis

For each patient, the unbound plasma concentration of phenytoin (Cu PHT) was calculated based on the measured total plasma concentration of phenytoin (Cp PHT) and the serum albumin concentration (ALB), in grams/decilitre, by using the original Winter-Tozer equation^5^ (Equation 1). The therapeutic range for the total phenytoin plasma concentration is 10 μg/mL to 20 μg/mL, and 1 μg/mL to 2 μg/mL for the unbound phenytoin plasma concentration:^4,5^


$$Cu\;PHT=\frac{Cp\;PHT}{\left(0.2\times ALB+0.1\right)}$$ [Eqn 1]

The unbound plasma fraction of valproic acid (α *VPA*) was calculated based on the serum albumin concentration (*ALB*) in μmol/L, by use of the hyperbolic equation proposed by Parent et al.^16,17^ (Equation 2):


$$\alpha \;VPA=130.69\times e^{-0.00496\times ALB}$$ [Eqn 2]

The unbound plasma concentration of valproic acid (Cu *VPA*) was then estimated for each patient based on the measured total valproic acid plasma concentration (Cp *VPA*) (Equation 3). The therapeutic ranges were 50 μg/mL to 100 μg/mL for total valproic acid plasma concentration and 2.5 μg/mL to 20.0 μg/mL for unbound valproic acid plasma concentration:^4,5^


Cu VPA=Cp VPA×α VPA [Eqn 3]

Descriptive statistics were used to summarise the results. All statistical calculations were carried out using the Excel software package of Windows 10 (Microsoft Corporation, Redmond, Washington, United States).

## Results

In total, 1661 files of patients hospitalised between January 2013 and July 2019 at the ICU of Windhoek Central Hospital were reviewed. Fifty-six patients, given phenytoin and 93 patients given valproic acid, who had data recorded for total plasma concentrations and serum albumin levels, were included in the study. Patients were admitted to the ICU for the following conditions: head trauma, hypoxic brain injury, infectious diseases such as meningitis and acute gastroenteritis, renal failure, and hepatic failure. The age of the patients ranged from two months to 66 years. The mean serum albumin levels were below 35 g/L in both sets of patients (mean ± s.d.: 23.5 ± 5.00 g/L for phenytoin, mean ± s.d.: 23.8 ± 5.9 g/L for valproic acid) (Table 1). Forty-five percent of patients on phenytoin were also receiving drugs known to interfere with its plasma binding or metabolism. For valproic acid, 28% of patients were concomitantly treated with drugs interfering with its plasma binding or metabolism.

**TABLE 1:** Patient characteristics and total and predicted plasma concentrations of phenytoin and valproic acid in critically ill patients in the Intensive Care Unit at Windhoek Central Hospital (Windhoek, Namibia) from 2013 to 2019.

Patient characteristics	Phenytoin (*n* = 56)				Valproic acid (*n* = 93)			
	*n*	%	Mean	Range	*n*	%	Mean	Range
**Patients**								
Male	29	-	-	-	49	-	-	-
Female	27	-	-	-	44	-	-	-
Age	-	-	23.3	2 months – 66 years	-	-	21.6	2 months – 63 years
Serum albumin (g/L)	-	-	23.5	14–34			23.8	9–39
Total concentration (μg/mL)†	-	-	12.6	0.1–43.0			32.5	2–75
Unbound concentration (μg/mL)‡	-	-	2.8	0.01–9.6			7.5	0.5–32.7
**Diagnosis**								
Head trauma	12	21.4	-	-	40	43.0	-	-
Hypoxic brain injury	-	-	-	-	11	11.8	-	-
Infectious diseases§	13	23.2	-	-	10	10.8	-	-
Renal failure/Hepatic failure	9	16.1	-	-	17	18.3	-	-
Neurological diseases (including epilepsy)	26	46.4	-	-	29	31.2	-	-
Patients receiving drugs known to interfere with plasma binding or metabolism of the drug	25	45.0	-	-	26	28.0	-	-

† , Therapeutic range: 10 μg/mL – 20 μg/mL (phenytoin) and 50 μg/mL – 100 μg/mL (valproic acid);

‡ , Therapeutic range: 1 μg/mL – 2 μg/mL for phenytoin, but not well defined for valproic acid;

§ , Tuberculosis (meningitis), pneumonia (bacterial meningitis).

**FIGURE 1:**
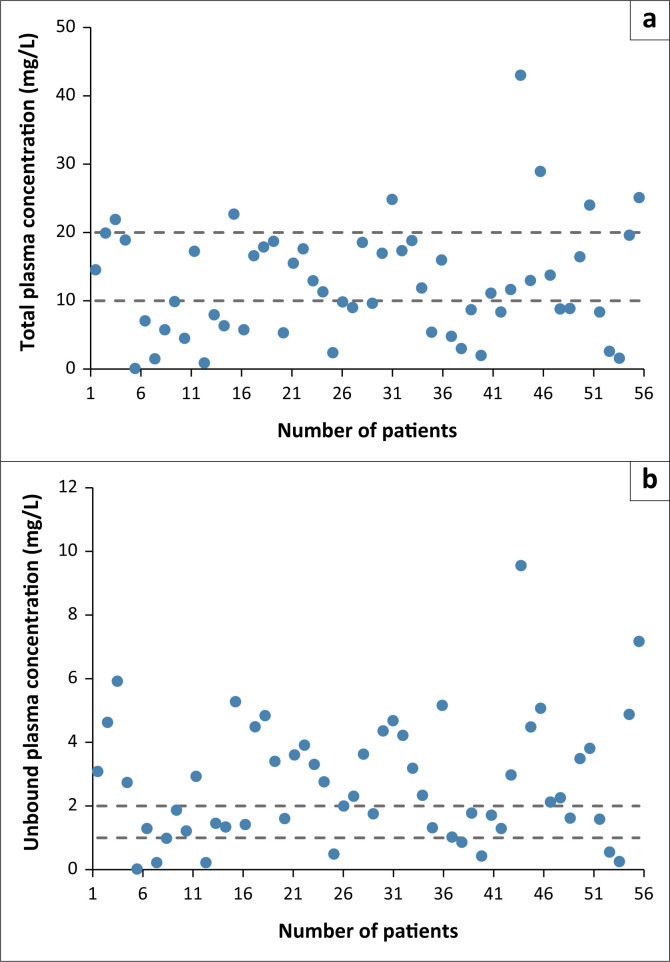
Plasma concentrations of (a) total and (b) unbound phenytoin plasma concentration in 56 critically ill patients in the Intensive Care Unit at Windhoek Central Hospital (Windhoek, Namibia) from January 2013 to July 2019. The therapeutic range is indicated by dashed lines.

The total plasma concentration of phenytoin recorded for 56 patients ranged from 0.1 μg/mL to 43.0 μg/mL, with an average of 12.6 ± 8.20 μg/mL (Table 1). In 23 patients (41.1%), the phenytoin total plasma concentration was within the therapeutic range (10 μg/mL to 20 μg/mL) (Figure 1). In 26 patients (46.4%), the plasma concentrations were subtherapeutic, that is, below 10 μg/mL, and in seven patients (12.5%) they were above 20 μg/mL. In 16 patients (28.6%), the predicted unbound plasma concentration of phenytoin was within the therapeutic range (1 μg/mL to 2 μg/mL). The unbound plasma concentration of phenytoin was subtherapeutic in eight patients (14.3%) and supratherapeutic in 32 patients (57.1%).

**FIGURE 2:**
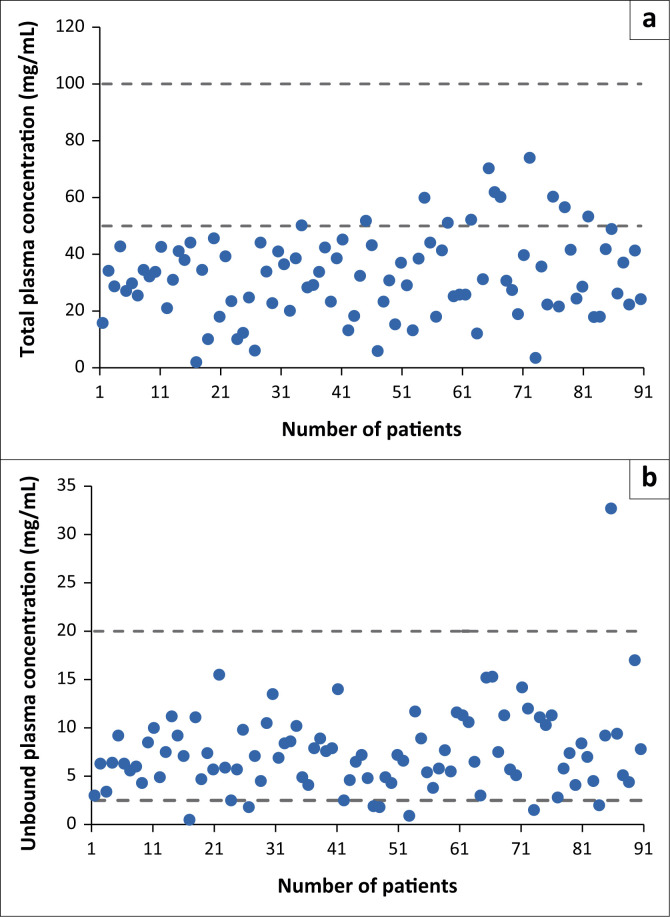
Plasma concentrations of (a) total and (b) unbound valproic acid plasma concentration in 93 critically ill patients in the Intensive Care Unit at Windhoek Central Hospital (Windhoek, Namibia) from January 2013 to July 2019. The therapeutic range is indicated by dashed lines. The suggested therapeutic range for unbound valproic acid concentrations is quite wide because the lower and upper limits of therapeutic ranges reported in the literature differ substantially.

For valproic acid, in 81 patients (87.1%), the total plasma concentration was below the therapeutic range of 50 μg/mL to 100 μg/mL (Figure 2). Binding of valproic acid to albumin in individual patients ranged from 9.0% to 39.0%. In 12 patients (12.9%), the total plasma concentration was within the therapeutic window, and no patients had a total plasma concentration of valproic acid above the therapeutic range. The average estimated unbound valproic acid plasma concentration was 24.0% (7.0% to 67.0%). In the majority of patients (*n* = 85, 91.4%), the unbound plasma concentration of valproic acid was within the therapeutic range (2.5 μg/mL to 20 μg/mL). In seven patients (7.5%), the unbound plasma concentration was below the therapeutic range, and in only one patient (1.1%) was it above the therapeutic range.

## Discussion

This retrospective study determined the plasma concentrations of phenytoin and valproic acid in critically ill patients at the ICU of Windhoek Central Hospital and found that the estimated unbound plasma concentration is a more accurate measure of the therapeutic plasma levels for the two drugs compared to total plasma concentration. Using the total plasma concentration, most patients were within or below the phenytoin therapeutic range. In contrast, unbound plasma concentration showed that less than one-third of the patients were within the therapeutic range and over half were above. For valproic acid, total plasma concentrations placed most patients were below the therapeutic range. However, unbound plasma concentrations placed majority within the therapeutic range.

Noval et al. reported a 2-fold increase in critical care patients having phenytoin concentrations falling within the therapeutic range when unbound concentrations instead of total phenytoin concentrations were considered for dose adjustment.^23^ In our study, the percentage of patients within therapeutic concentrations using total plasma concentrations reduced from 41.1% to 28.6% when an unbound phenytoin concentration was used. Thus, the use of total phenytoin concentrations suggests that dosing in these patients was at target therapeutic concentrations, suggesting no optimal patient dosage. The high variability in the binding of valproic acid to albumin observed in this study (9.0% – 39.0%) reflects reports in the literature, such as the 10.0% – 60.0% range reported in ICU patients by Lagneau et al.^13^ and the 15.0% – 89.0% range reported by Riker et al.^24^ This high variability in plasma protein binding explains the marked difference in the percentage of patients having concentrations that were within the therapeutic range when total valproic acid was considered (12.9%) compared to when the unbound valproic acid concentrations were taken into account (91.4%). Total valproate concentration is a poor predictor of the unbound drug concentration, even when correction is made for albumin.^13^

Therapeutic drug monitoring of phenytoin and valproic acid by measuring unbound plasma concentrations is not widespread because of the additional time and cost implications. Thus, some therapeutic drug monitoring services measure only the total drug plasma concentrations or estimate the unbound plasma drug concentrations. These equations, including those used in this study, estimate the unbound concentrations of phenytoin and valproic acid based on the patient’s measured total drug plasma concentration and serum albumin level.^5,18,19,20,21,22^ Unfortunately, these equations are not very accurate and may underestimate the unbound plasma concentrations of phenytoin and valproic acid in critically ill patients.^25,26,27,28,29,30^ In addition, although the therapeutic plasma range for unbound phenytoin is relatively established (1 μg/mL – 2 μg/mL), various therapeutic ranges for the unbound plasma concentration of valproic acid have been proposed in the literature. However, more research is needed to determine the optimal unbound plasma concentration range for this anticonvulsant.^31^ Furthermore, recent investigations on the predictive performance of these equations led to the conclusion that more complex, multivariate predictive equations may be required, which, in addition to the total drug plasma concentration and serum albumin level, considering the ICU status of the patient, age, and blood urea nitrogen level.^30,28^

Therapeutic drug monitoring of phenytoin and valproic acid should be based on unbound drug concentrations. The best way to monitor the unbound drug concentration would be by measuring it directly in ultrafiltrate obtained from serum to ensure that the accurate concentration of the free drug is determined and used to inform dosage adjustment.

### Limitations

The main limitation of this retrospective study is that the unbound plasma concentrations of phenytoin and valproic acid were not determined experimentally. However, because the albumin-based normalising equations that were used underestimate the patient’s unbound plasma concentrations of phenytoin and valproic acid, the discordance between total and unbound concentrations is less pronounced than it would be in reality. In addition, there was no follow-up to adjust the dosage regimen of these two drugs in the individual patients based on the observed total plasma concentrations.

### Conclusion

In conclusion, for therapeutic drug monitoring of phenytoin and valproic acid in critically ill patients, direct measurement of the unbound phenytoin and valproic acid concentration in plasma would be the best approach.
